# Ten‐Year Split‐Mouth Follow‐Up of Tunnel Technique Using Connective Tissue Graft Versus Acellular Dermal Matrix for Multiple Gingival Recession Coverage: A Case Report

**DOI:** 10.1155/crid/1995303

**Published:** 2026-10-23

**Authors:** Roberto Ledergerber, Matthew Lama, Antonio Gabriel Lanata-Flores

**Affiliations:** ^1^ School of Dentistry Universidad de Especialidades Espíritu Santo (UEES) Samborondón Ecuador

**Keywords:** acellular dermal matrix, connective tissue transplantation, esthetics dental, follow-up studies, gingival recession, periodontal plastic surgery

## Abstract

**Introduction:**

Long‐term split‐mouth comparisons between autogenous connective tissue grafts and acellular dermal matrices using the tunnel technique remain limited in the literature. Gingival recession may lead to root exposure, hypersensitivity, and esthetic concerns. Connective tissue grafts are widely considered the gold standard for root coverage; however, palatal harvesting is associated with postoperative morbidity when multiple sites are treated. Acellular dermal matrix has been proposed as a donor‐site‐free alternative.

**Case Presentation:**

A 55‐year‐old female patient presented with multiple RT1 gingival recessions and dentin hypersensitivity. A split‐mouth design was performed. On the right maxilla, a tunnel technique combined with an autogenous connective tissue graft was used, whereas on the left maxilla, the same technique was combined with acellular dermal matrix. Clinical measurements and photographic documentation were obtained up to 10 years.

**Results:**

During the first 2 years, mean root coverage was 100% on the CTG‐treated side and 95% on the ADM‐treated side. At Year 6, mean root coverage remained 100% on the CTG‐treated side and decreased to 67% on the ADM‐treated side. Complete root coverage was maintained in 75% and 40% of sites, respectively. Relapse occurred primarily in acellular dermal matrix–treated teeth after Year 6, whereas the soft‐tissue phenotype remained clinically stable on both sides. The patient reported high satisfaction and complete resolution of hypersensitivity.

**Conclusion:**

In this single case, the CTG‐treated side showed greater observed stability at 10 years, whereas ADM provided satisfactory clinical outcomes with lower donor‐site morbidity. These observations are descriptive and should not be interpreted as evidence of comparative effectiveness or generalized beyond this patient.

## 1. Introduction

Gingival recession is defined as the apical displacement of the gingival margin relative to the cementoenamel junction (CEJ), resulting in root exposure that may lead to hypersensitivity, as well as esthetic and functional alterations. Its prevalence increases with age and with the presence of local predisposing factors, such as a thin periodontal phenotype, toothbrushing trauma, and controlled periodontal disease. In addition, it can be observed in individuals with either good or poor oral hygiene [1].

Root hypersensitivity can be managed through several noninvasive approaches such as desensitizing varnishes or composite restorations; however, in patients with high smile lines, esthetic concerns often necessitate gingival tissue regeneration to achieve satisfactory results [1].

Root coverage procedures have evolved from free gingival grafts and staged coronally positioned flaps to more refined flap designs intended to improve esthetic outcomes [2]. The coronally advanced flap (CAF) described by Zucchelli and De Sanctis avoids vertical releasing incisions and facilitates the treatment of multiple adjacent recessions [3]. Among the available grafting materials, subepithelial connective tissue graft (CTG) remains widely regarded as the reference material because of its documented root coverage, keratinized tissue (KT) gain, and long‐term stability [4, 5].

Long‐term clinical studies indicate that adding CTG to a CAF increases the probability of complete root coverage (CRC) and improves gingival margin stability compared with the flap alone [4, 5]. Nevertheless, harvesting tissue from the palate introduces disadvantages, including postoperative discomfort, bleeding risk, and limitations in the amount of donor tissue available when several sites require treatment [6].

To overcome these drawbacks, alternative biomaterials have been developed. Among them, acellular dermal matrix (ADM) is a collagen that preserves the dermal extracellular architecture, allowing revascularization and tissue integration while eliminating the need for a donor site [7, 8].

In parallel, less invasive surgical approaches have been proposed, such as the tunnel technique (TUN) described by Zabalegui et al. [9], based on the supraperiosteal envelope concept introduced by Allen in 1994 [10]. These techniques avoid vertical incisions, preserve interdental papillae, and optimize graft perfusion, promoting faster healing and improved esthetic outcomes.

Long‐term studies have reported satisfactory outcomes following root coverage procedures involving CTG or ADM used with CAF or TUN [11–13]. Nevertheless, apical displacement of the gingival margin and partial loss of root coverage have been documented after 5–12 years [6, 7, 14, 15]. The biological and local factors responsible for this recurrence remain incompletely understood.

In this context, the present case report with a 10‐year follow‐up compares two surgical approaches in a split‐mouth design:

• TUN + CTG, as the conventional autogenous technique, and
• TUN + ADM, as a minimally invasive, donor‐site‐free alternative.

The objective was to evaluate the long‐term clinical and esthetic stability of both techniques, analyzing soft tissue healing, gingival margin stability, and patient satisfaction after a decade of continuous follow‐up.

This case report was prepared in accordance with the CARE guidelines (File S1).

## 2. Case Presentation

A 55‐year‐old female patient (ASA I) presented with multiple RT1 gingival recessions associated with dentin hypersensitivity. Based on the patient′s history and clinical examination, traumatic toothbrushing was considered the primary etiologic factor, whereas a thin periodontal phenotype was identified as an important predisposing factor. Before surgery, the patient received oral hygiene counseling aimed at reducing toothbrushing‐related trauma. These recommendations were reinforced during follow‐up and supportive periodontal maintenance visits. The thin periodontal phenotype was addressed through soft‐tissue augmentation using CTG on one side and ADM on the contralateral side. The patient sought treatment at a private periodontal clinic, where a comprehensive clinical and radiographic evaluation was performed, including documentation of recession depth (RD), clinical attachment level (CAL), bleeding on probing, and gingival phenotype. The patient reported having received previous dental restorations, with no history of surgical treatment. A recession coverage procedure using an autologous CTG was initially proposed. The treatment plan and alternatives were thoroughly explained, and the patient provided written informed consent before proceeding. All surgeries and measurements were performed by the same clinician. Baseline photographs illustrating initial gingival recessions are shown (Figure 1A–C). Full‐mouth periodontal charting was performed at baseline and at the 10‐year follow‐up, including RD, probing depth (PD), CAL, and KT width. RD was additionally recorded at the 3–6‐month, 1‐year, 2‐year, 6‐year, 7‐year, 9‐year, and 10‐year evaluations. Measurements were obtained at the midbuccal aspect of each treated tooth using a calibrated periodontal probe and consistent anatomical reference points.

**Figure 1:**
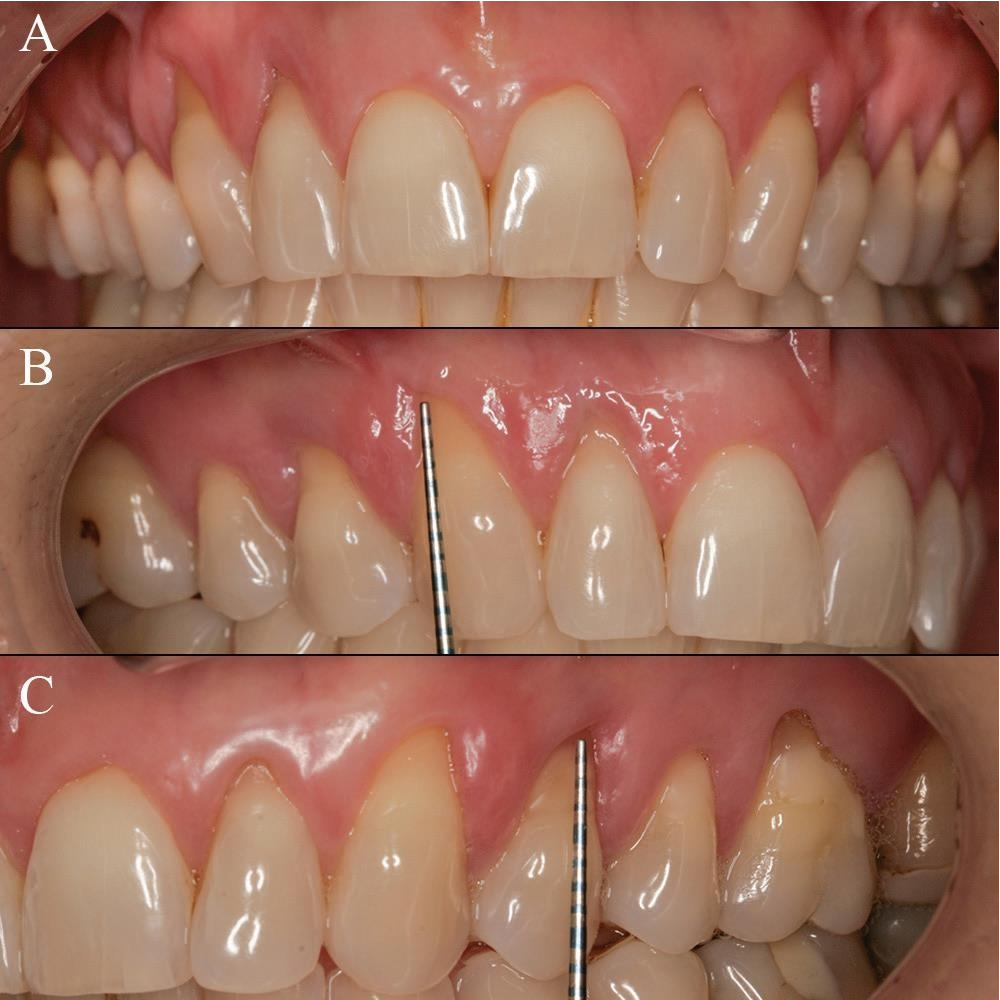
(A) Preoperative full view showing multiple maxillary gingival recessions. (B) Baseline view of the right maxilla. (C) Baseline view of the left maxilla.

RD was defined as the distance from the CEJ to the gingival margin. CAL was measured from the CEJ to the base of the sulcus, PD from the gingival margin to the bottom of the sulcus, and KT from the gingival margin to the mucogingival junction. Mean root coverage (MRC) was calculated separately for the CTG‐ and ADM‐treated sides, whereas CRC was defined as the absence of residual recession. Measurements obtained at the different follow‐up points are presented in Table 1.

**Table 1:** Mean root coverage over time according to the treated side.

Follow‐up time point	Right side (TUN + CTG), MRC (%)	Left side (TUN + ADM), MRC (%)
Baseline	0	0
3–6 months	100	95
1 year	100	95
2 years	100	95
6 years	100	67
7 years	100	67
9 years	100	67
10 years	90	67

Abbreviations: ADM, acellular dermal matrix; CTG, connective tissue graft; MRC, mean root coverage; TUN, tunnel technique.

Esthetic outcomes were assessed descriptively through clinical examination and serial intraoral photographs, considering gingival margin position, tissue contour, color, texture, visible scarring, and integration with adjacent tissues. Dentin hypersensitivity and treatment satisfaction were documented according to the patient′s reports during follow‐up visits. No validated questionnaire or quantitative esthetic or hypersensitivity scale was used; therefore, these outcomes are reported descriptively.

### 2.1. First Surgery: TUN With CTG

A TUN was performed from Teeth #11 to #16. Following intrasulcular incisions, a full‐thickness envelope flap was elevated through the sulcus using blunt periotomes from the PPAEL Periotomes Kit up to 2 mm beyond the mucogingival junction [9, 10]. A split‐thickness dissection was then extended apically approximately 15 mm using a tunneling blade until complete tunnel release was achieved. The interdental papillae were detached full thickness to allow adequate coronal mobilization, as described by Allen [10].

A subepithelial CTG was harvested from the palatal premolar region using the single‐incision technique [16] to minimize morbidity. The graft was inserted into the tunnel and stabilized with single interrupted sutures. The harvested CTG measured approximately 20 mm in length, 4 mm in height, and 1.5 mm in thickness. The graft was inserted as a single uniform block covering all treated teeth. A continuous sling suture using 5‐0 polypropylene monofilament was placed to coronally advance the flap, leaving approximately 1–2 mm of the graft exposed [10].

Postoperative instructions included avoiding brushing in the treated area, maintaining a soft diet, and abstaining from physical exercise for 1 week. Medications included ibuprofen 600 mg and acetaminophen 1 g every 8 h for 5 days, and amoxicillin 500 mg every 8 h for 7 days. The palatal donor site was sutured to allow healing by primary intention. Postoperative instructions were reinforced, and healing was uneventful at both donor and recipient sites. Sutures were removed after 3 weeks. The first procedure using CTG on the right side is illustrated in Figure 2A,B.

**Figure 2:**
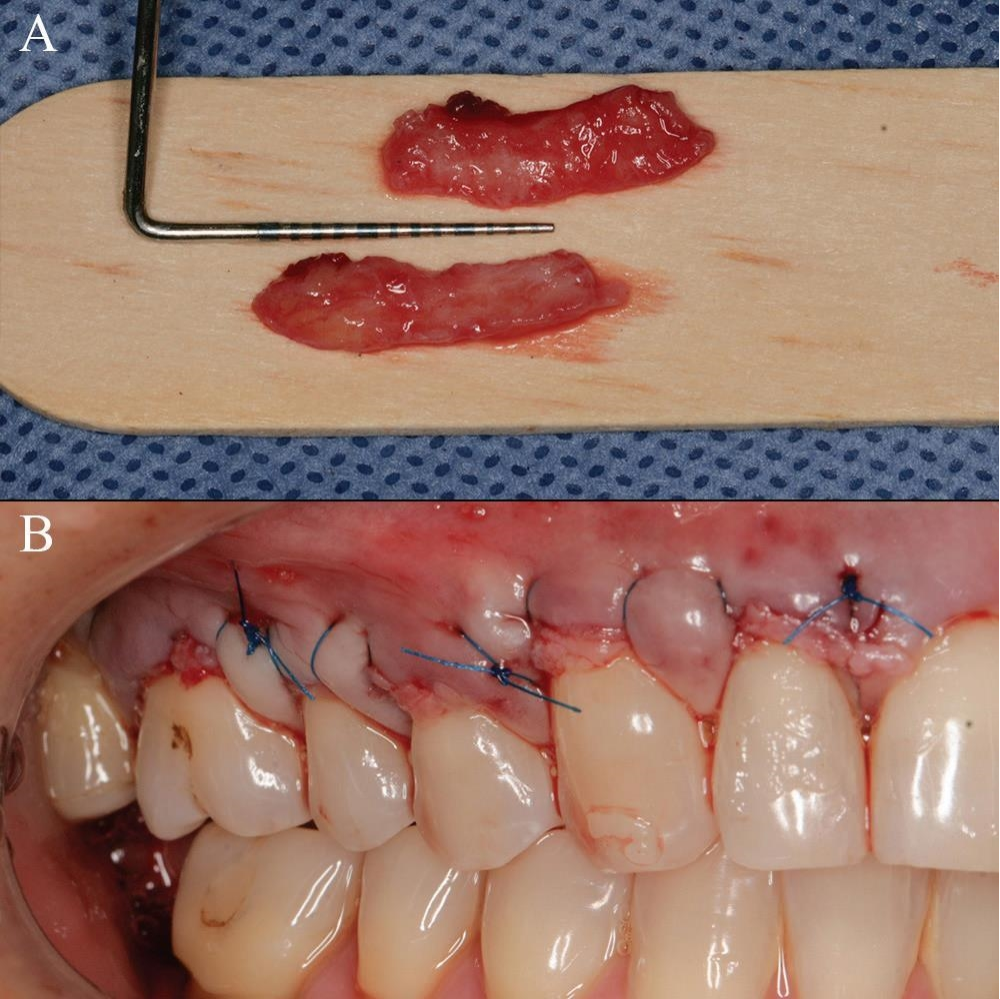
(A) Harvested subepithelial connective tissue from the palate. (B) Tunnel advancement and graft stabilization with sling sutures.

### 2.2. Second Surgery: TUN With ADM

Three months later, a second tunnel procedure was performed on the contralateral side (#21–#27). The patient declined another palatal graft harvest and opted for the use of an ADM [17].

Before surgery, existing composite restorations on Teeth #24–#26 were polished and coronally recontoured to harmonize the restorative margins with the intended gingival position. Noncarious cervical lesions (NCCLs) were present on Teeth #12, #24, #25, and #26; Teeth #24–#26 had been restored with composite resin prior to surgery. The surgical technique was performed according to the same clinical principles as the first procedure, with slight modifications related to the graft material [9, 10]. After flap preparation, the ADM was rehydrated, trimmed with rounded edges, and prepared as a single block with a standardized height of approximately 7–8 mm [8, 17]. The graft was inserted into the tunnel and stabilized mesially and distally with single interrupted sutures. A continuous sling suture using 5‐0 nylon monofilament was used to coronally advance the flap, fully covering the graft and positioning the marginal tissues approximately 1–2 mm coronal to the desired final gingival margin, as described by Allen [10]. Although both procedures used the same suture diameter, the materials differed: Polypropylene was used on the CTG‐treated side and nylon on the ADM‐treated side. The greater stiffness of nylon compared with polypropylene represents an additional technical difference between the procedures.

Postoperative care followed the same regimen as the previous surgery. Healing was uneventful, and sutures were removed after 3 weeks. The subsequent ADM procedure on the left maxilla is presented in Figure 3A,B.

**Figure 3:**
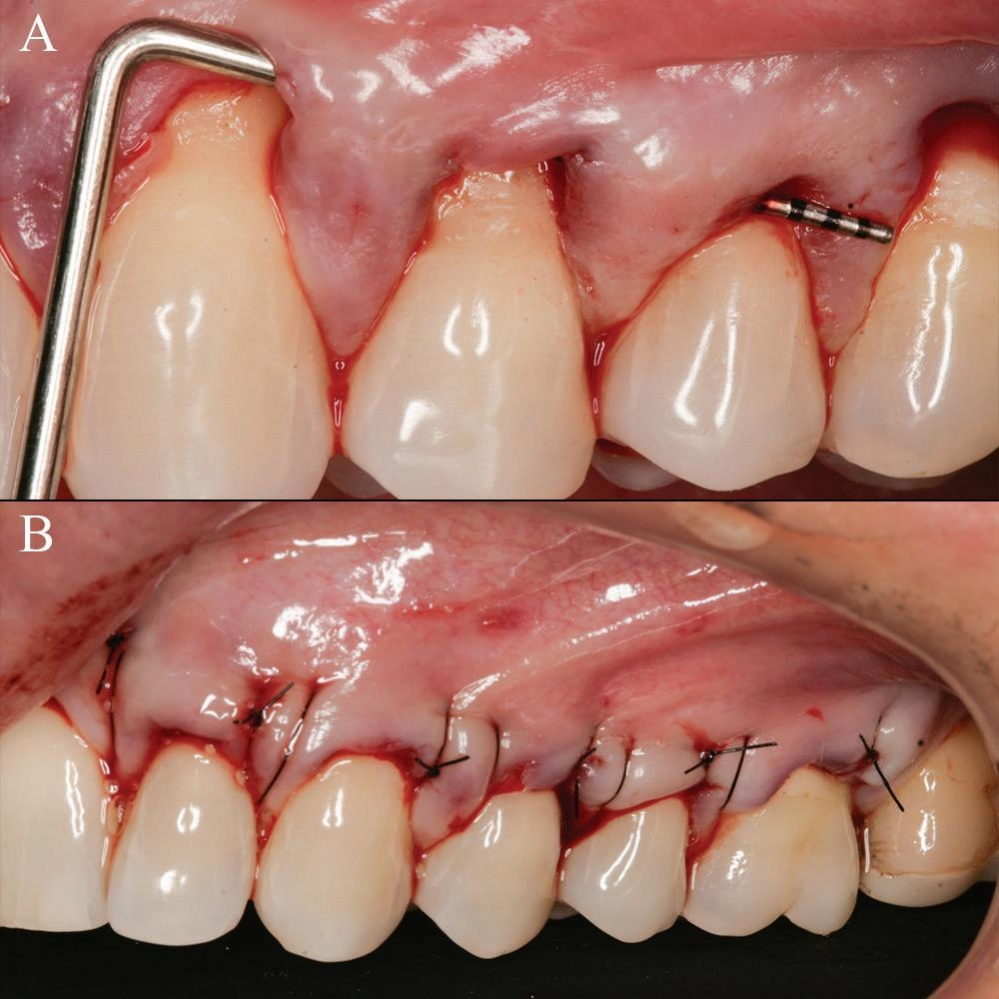
(A) Tunnel preparation on the left maxilla. (B) Tunnel advancement and ADM positioned and sutured in place.

### 2.3. Follow‐Up and Supportive Periodontal Care

The patient attended supportive periodontal maintenance visits twice per year throughout the follow‐up period. Each visit included professional prophylaxis, evaluation of plaque control and oral hygiene practices, and reinforcement of the oral hygiene recommendations. The patient demonstrated consistent compliance, and full‐mouth plaque and gingival indices remained below 15% throughout the follow‐up period.

Clinical measurements were performed by the same clinician using a calibrated periodontal probe and consistent anatomical reference points. Because the measurements were obtained during routine clinical care, no formal intraexaminer reliability coefficient was available. Serial photographs were obtained at the principal follow‐up points using comparable clinical views; however, a formally standardized photographic protocol was not documented. The schedule of clinical measurements and root coverage assessments is presented in Table 1.

## 3. Results

The patient was evaluated at 3 weeks, 3 months, 6 months, and annually thereafter. Clinical photographs and measurements were recorded up to 10 years postoperatively.

### 3.1. Clinical Outcomes

On the right maxilla, a TUN with an autologous CTG was performed; on the left maxilla, the same technique was combined with an ADM.

At baseline, RDs ranged from 1 to 3 mm on the CTG side and from 1 to 4 mm on the ADM side (Table 1). PDs remained ≤ 3 mm at baseline and at the 10‐year follow‐up, with no clinical signs of bleeding on probing observed during maintenance visits.

Both procedures achieved 100% MRC during the first 6 years, with stable soft‐tissue thickness and a visibly thickened phenotype, except for Tooth #26, which exhibited 75% root coverage immediately after surgery. From Years 7 to 10, minor changes were observed:

• CTG side (Teeth #12–#15): maintained 100% coverage for all sites, except Tooth #13, which presented a 1 mm relapse at Year 9 corresponding to a −60% change relative to the initial 100% MRC.
• ADM side (Teeth #22–#26): Teeth #22 and #23 remained fully covered through 10 years. In contrast, Teeth #24–#26 showed gradual recession beginning at Year 6, with final coverage ranging between −33% and −50% at Year 10.

After 10 years, MRC values were 90% for the CTG group and 67% for the ADM group. CRC was observed in 75% of the CTG sites and in 40% of the ADM sites. MRC trends for the CTG‐ and ADM‐treated sides across the follow‐up time points are summarized in Table 2.

**Table 2:** Long‐term evolution of midbuccal recession depth (RD) and mean root coverage (MRC) at treated sites.

Tooth	Intervention	Baseline RD (mm)^a^	3–6 months (RD, MRC)^b^	1 year (RD, MRC)^b^	2 years (RD, MRC)^b^	6 years (RD, MRC)^b^	7 years (RD, MRC)^b^	9 years (RD, MRC)^b^	10 years (RD, MRC)^b^
12	CTG	−2	0, 100%	100%	100%	100%	100%	100%	100%
13	CTG	−3	0, 100%	100%	100%	100%	100%	100%	−1, 60%
14	CTG	−2	0, 100%	100%	100%	100%	100%	100%	100%
15	CTG	−2	0, 100%	100%	100%	100%	100%	100%	100%
22	ADM	−1	0, 100%	100%	100%	100%	100%	100%	100%
23	ADM	−2	0, 100%	100%	100%	100%	100%	100%	100%
24	ADM	−3	0, 100%	100%	100%	−2, 33%	−2, 33%	−2, 33%	−2, 33%
25	ADM	−2	0, 100%	100%	100%	−1, 50%	−1, 50%	−1, 50%	−1, 50%
26	ADM	−4	−1, 75%	−1, 75%	−1, 75%	−2, 50%	−2, 50%	−2, 50%	−2, 50%

Abbreviations: ADM, acellular dermal matrix; CTG, connective tissue graft; MRC, mean root coverage; RD, recession depth.

^a^RD was recorded at the midbuccal site and expressed relative to the CEJ; negative values indicate an apical position of the gingival margin relative to the CEJ.

^b^For follow‐up columns, values are presented as “RD (millimeter), MRC (%).” An RD of 0 mm corresponds to 100% root coverage; residual or recurrent recession is shown as a negative RD value with the corresponding MRC percentage.

Figures 4 and 5 show early healing and CRC at 6 months and 2 years. Figure 6 depicts midterm outcomes at 6 years, highlighting the onset of relapse on the ADM side, whereas Figure 7 presents the 10‐year long‐term follow‐up demonstrating overall stability. No adverse events or graft exposures occurred at any time. At Year 7, the composite restorations on Teeth #24–#26 were replaced after loss of retention associated with bonding failure and to manage the recurrent cervical root exposure. This restorative intervention was performed only on the ADM‐treated side, after marginal relapse had become clinically evident. No comparable restorative intervention was performed on the CTG‐treated side during the follow‐up period.

**Figure 4:**
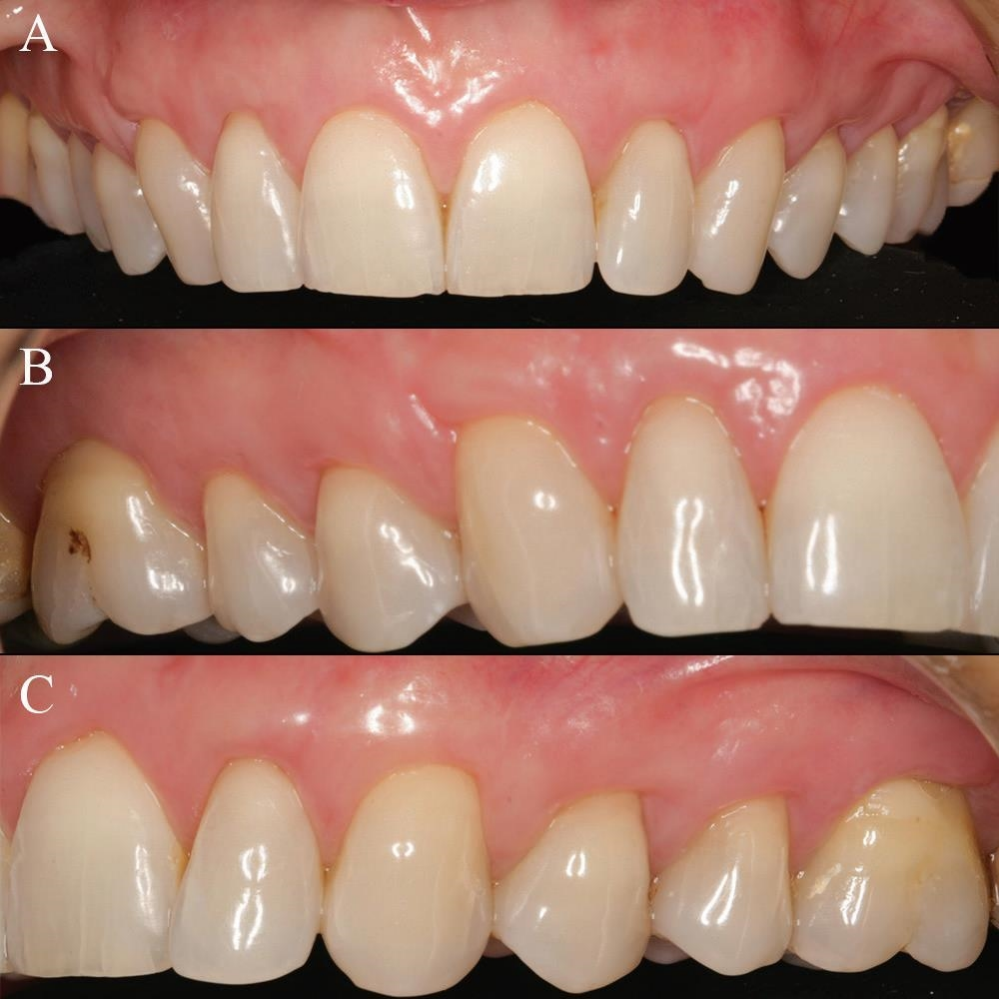
(A) Six‐month postoperative frontal view. (B) Right maxilla (CTG‐treated side). (C) Left maxilla (ADM‐treated side).

**Figure 5:**
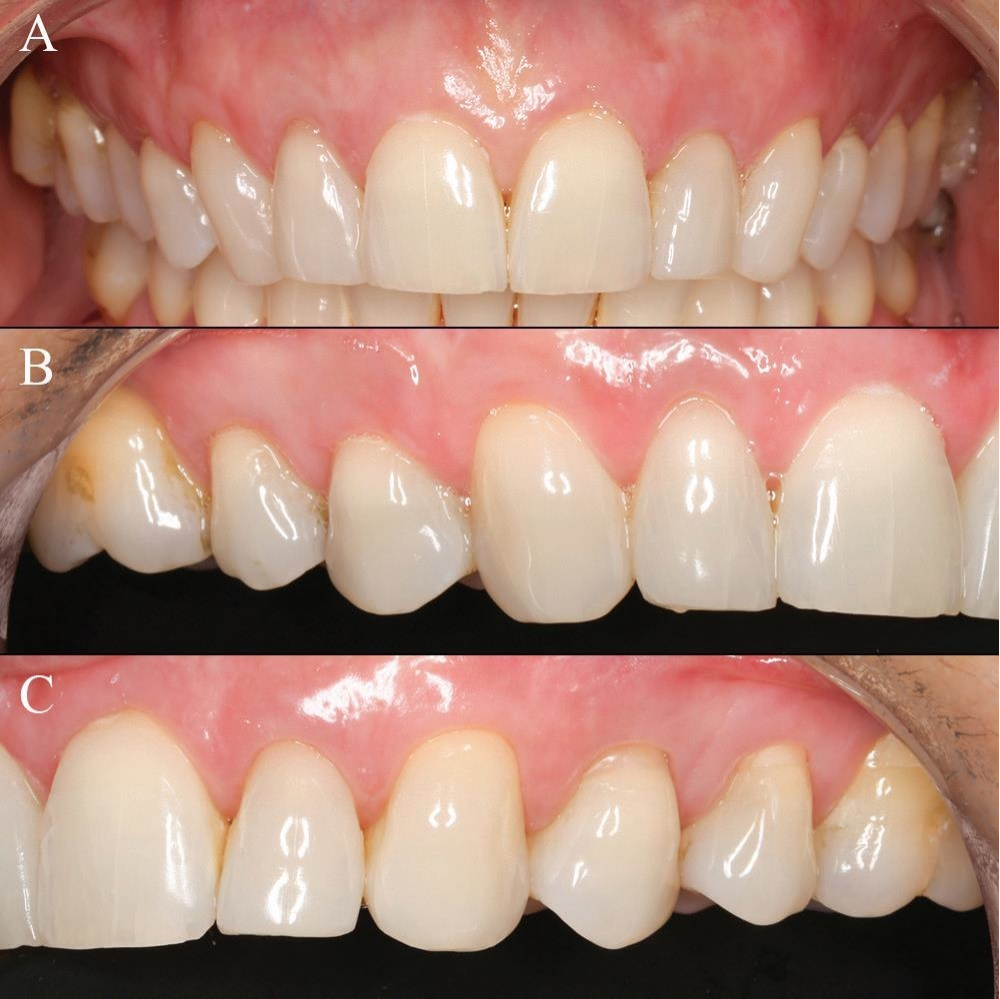
(A) Two‐year postoperative frontal view. (B) Right maxilla (CTG‐treated side). (C) Left maxilla (ADM‐treated side).

**Figure 6:**
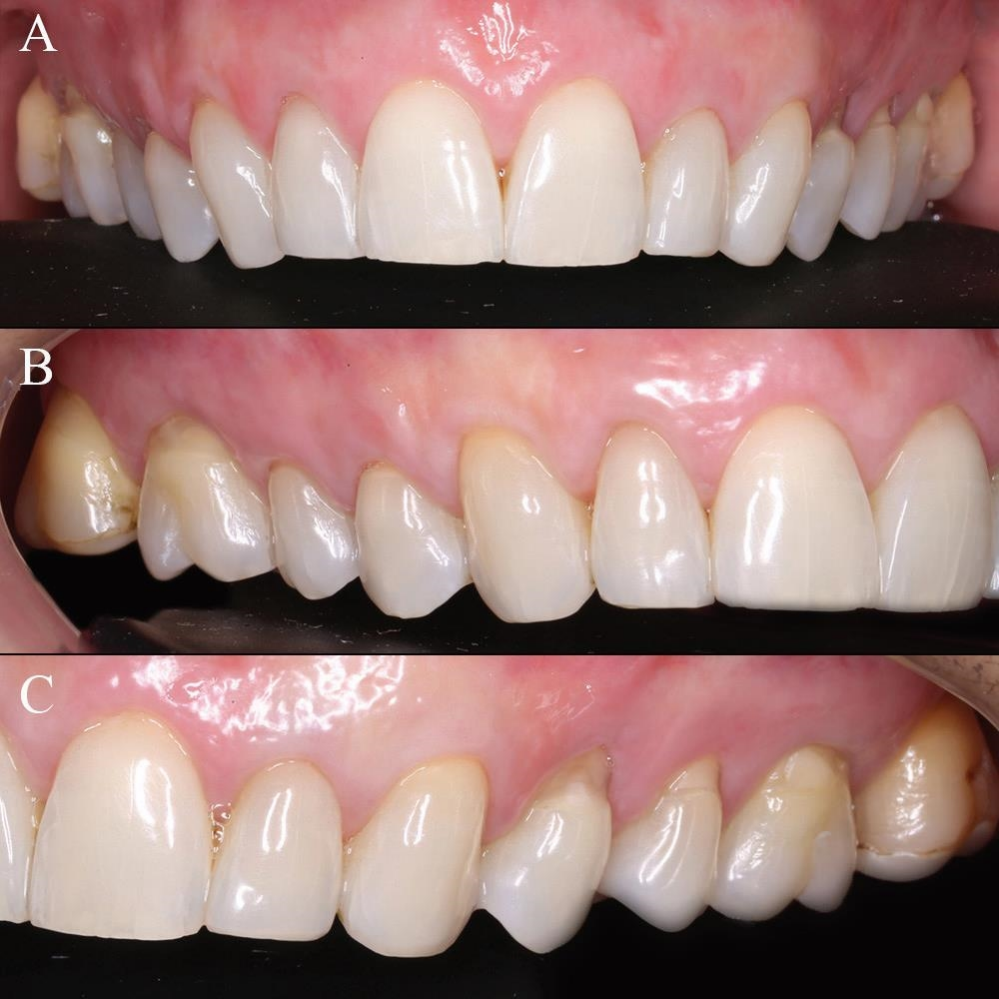
(A) Six‐year postoperative frontal view. (B) Right maxilla (CTG‐treated side) showing stable coverage. (C) Left maxilla (ADM‐treated side) showing partial marginal relapse.

**Figure 7:**
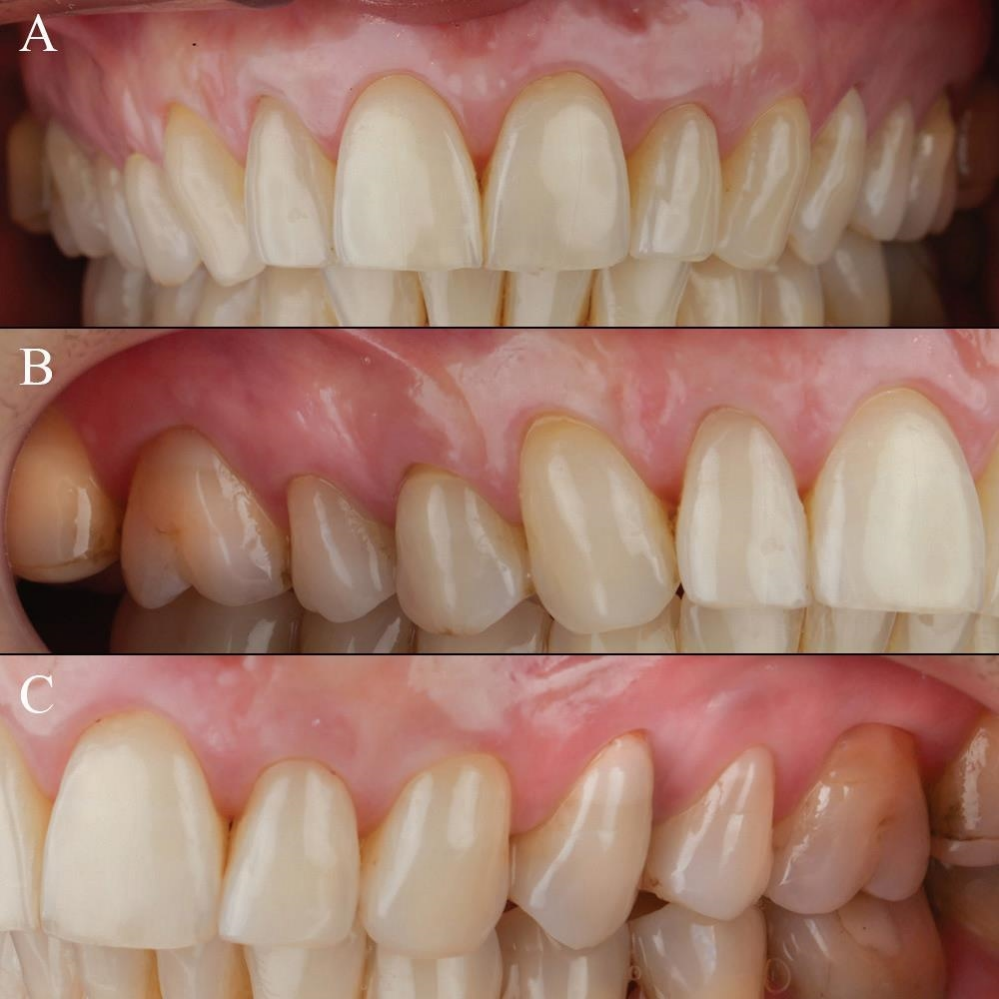
(A–C) Ten‐year postoperative views showing long‐term stability of the CTG‐treated side (right maxilla) and partial relapse on the ADM‐treated side (left maxilla).

### 3.2. Patient‐Reported Outcomes

The patient reported high satisfaction with esthetics and function for both procedures. Hypersensitivity resolved completely after surgery and did not recur. Despite slightly greater long‐term stability on the CTG side, the patient expressed a clear preference for the ADM technique due to reduced postoperative morbidity and absence of palatal discomfort.

### 3.3. Soft‐Tissue Clinical Appearance

Serial clinical examinations and intraoral photographs showed maintenance of tissue contour, color, texture, and integration with the adjacent tissues at both treated sites, without visible scarring. The band of KT remained clinically evident at the final evaluation, although marginal relapse was observed at several teeth on the ADM‐treated side.

Gingival thickness was not quantitatively assessed using transgingival probing, ultrasonography, or another standardized measurement method. Therefore, no comparison of tissue thickness between the CTG‐ and ADM‐treated sides can be made. The reported soft‐tissue findings are limited to descriptive clinical and photographic observations.

## 4. Discussion

This case illustrates successful root coverage using the tunnel approach combined with either a CTG or a dermal matrix after 10 years of follow‐up, demonstrating long‐term stability of soft‐tissue integration. Both techniques yielded comparable outcomes, with a slightly superior long‐term result observed for the CTG.

Systematic reviews support CTG‐based procedures as a predictable option for root coverage and recognize ADM as a possible alternative to autogenous donor tissue [12, 13]. However, the systematic review by Chambrone and Tatakis does not establish the long‐term superiority of CTG over ADM [12]. Direct long‐term comparisons remain limited, and the available evidence does not permit a definitive hierarchy between these materials over extended follow‐up periods.

In contrast, MRC at 10 years was 90% on the CTG‐treated side and 67% on the ADM‐treated side, whereas CRC was observed in 75% and 40% of sites, respectively. These values describe the clinical evolution of this individual patient but should not be interpreted as evidence of material superiority because of the single‐patient design and the different local restorative conditions between sides [7, 18].

Although some reports have suggested that autogenous grafts may present more frequent soft‐tissue irregularities, scar formation, or lower pink‐esthetic scores (PESs) compared with dermal matrices, no soft‐tissue alterations were observed on the connective tissue side in this case [19–21]. This may be attributed to the use of a subepithelial connective tissue graft harvested through a single‐incision technique, which minimizes the risk of epithelial remnants that can lead to such complications [16].

Patient‐reported outcomes were positive for both approaches. The patient perceived similar esthetic and functional results on both sides up to the 6th year, after which occlusal problems, including muscle discomfort, a lower molar fracture, and progression of the cervical lesions, became evident. On the ADM‐treated side, marginal relapse occurred in the presence of restored NCCLs, subsequent bonding failure, and restorative replacement at Year 7. Previous studies have indicated that NCCLs and their restorative management may influence the outcomes of root coverage procedures [22, 23]. However, because the restorative conditions and interventions differed between sides, the relative contributions of the graft material, lesion progression, bonding failure, and restorative instrumentation cannot be determined. Therefore, no conclusion regarding greater protection by either graft material can be drawn from this case.

Short‐term outcomes (6 months) are illustrated in Figure 4A–C, midterm outcomes at 2 and 6 years, including onset of relapse on the ADM side, are shown in Figures 5A–C and 6A–C, respectively, and long‐term stability at 10 years is shown in Figure 7.

This case reinforces the long‐term reliability of the TUN combined with connective tissue grafting as the gold standard for root coverage, while highlighting that dermal matrices represent a valuable, less invasive alternative capable of delivering satisfactory clinical and patient‐reported outcomes. Although long‐term stability continues to favor autogenous connective tissue, dermal matrices can still achieve esthetically pleasing and functionally stable results over several years [24]. Importantly, patient preferences played a decisive role: Despite the slightly superior results obtained with the connective tissue graft, the patient expressed a preference for the dermal matrix in future treatments due to reduced morbidity and greater postoperative comfort. This underscores the importance of patient‐centered decision‐making and the need to balance biological predictability with patient comfort and expectations. Clinicians should therefore consider both the scientific evidence supporting connective tissue grafts and the practical advantages of less invasive alternatives, tailoring treatment choices to individual patient needs and priorities.

The restorative intervention performed on Teeth #24–#26 represents an important local confounding factor. Replacement of the restorations required instrumentation exclusively on the ADM‐treated side after marginal relapse had become evident. Therefore, it is not possible to determine whether the subsequent clinical findings were associated with the graft material, progression of the NCCLs, bonding failure, restorative instrumentation, or a combination of these factors. The outcomes should consequently not be interpreted as a controlled comparison of CTG and ADM.

## 5. Conclusion

Within the limitations of this single‐patient case report, both TUN + CTG and TUN + ADM provided satisfactory clinical and patient‐reported outcomes. Greater observed stability was found on the CTG‐treated side over the 10‐year follow‐up. However, the single‐patient design and differences in local restorative conditions prevent attributing the observed outcomes exclusively to the graft materials. These findings are descriptive and highlight the need for controlled long‐term studies.

## Author Contributions

Roberto Ledergerber: conceptualization, methodology, resources, visualization, and supervision; Matthew Lama: formal analysis and writing—review and editing; Antonio Gabriel Lanata‐Flores: conceptualization, data curation, project administration, supervision, and writing—review and editing.

## Funding

No funding was received for this manuscript.

## Disclosure

All authors have read and approved the final version of the manuscript. Antonio Gabriel Lanata‐Flores had full access to all of the data in this study and takes complete responsibility for the integrity of the data and the accuracy of the data analysis.

## Ethics Statement

Formal ethical approval was not required for this single‐patient case report because it describes routine clinical care and involved no experimental intervention. Written informed consent was obtained from the patient for treatment, long‐term clinical follow‐up and data collection, and publication of the clinical information and images. The signed consent form is securely archived by the corresponding author.

## Conflicts of Interest

The authors declare no conflicts of interest.

## Supporting information


**Supporting Information** Additional supporting information can be found online in the Supporting Information section. File S1: Completed CARE checklist for this case report.

## Data Availability

The data that support the findings of this study are available on request from the corresponding author. The data are not publicly available due to privacy or ethical restrictions.

## References

[1] Cortellini P. and Bissada N. F. , Mucogingival Conditions in the Natural Dentition: Narrative Review, Case Definitions, and Diagnostic Considerations, Journal of Periodontology. (2018) 89, S204–S213, 10.1002/JPER.16-0671, 29926948. 29926948

[2] Bernimoulin J. P. , Lüscher B. , and Mühlemann H. R. , Coronally Repositioned Periodontal Flap. Clinical Evaluation After One Year, Journal of Clinical Periodontology. (1975) 2, no. 1, 1–13, 10.1111/j.1600-051X.1975.tb01721.x. 1055724

[3] Zucchelli G. and De Sanctis M. , Treatment of Multiple Recession-Type Defects in Patients With Esthetic Demands, Journal of Periodontology. (2000) 71, no. 9, 1506–1514, 10.1902/jop.2000.71.9.1506, 11022782. 11022782

[4] Pini-Prato G. , Franceschi D. , Rotundo R. , Cairo F. , Cortellini P. , and Nieri M. , Long-Term 8-Year Outcomes of Coronally Advanced Flap for Root Coverage, Journal of Periodontology. (2012) 83, no. 5, 590–594, 10.1902/jop.2011.110410, 22014171. 22014171

[5] Rasperini G. , Acunzo R. , Pellegrini G. , Pagni G. , Tonetti M. , Pini Prato G. P. , and Cortellini P. , Predictor Factors for Long-Term Outcomes Stability of Coronally Advanced Flap With or Without Connective Tissue Graft in the Treatment of Single Maxillary Gingival Recessions: 9 Years Results of a Randomized Controlled Clinical Trial, Journal of Periodontology. (2018) 45, no. 9, 1107–1117, 10.1111/jcpe.12932. 29777632

[6] Zuhr O. , Akakpo D. , Eickholz P. , Vach K. , Hürzeler M. B. , Petsos H. , and the Research Group for Oral Soft Tissue Biology & Wound Healing , Tunnel Technique With Connective Tissue Graft Versus Coronally Advanced Flap With Enamel Matrix Derivate for Root Coverage: 5-Year Results of an RCT Using 3D Digital Measurement Technology for Volumetric Comparison of Soft Tissue Changes, Journal of Clinical Periodontology. (2021) 48, no. 7, 949–961, 10.1111/jcpe.13470, 33847022. 33847022

[7] Tavelli L. , Barootchi S. , Cairo F. , Rasperini G. , Shedden K. , and Wang H. L. , The Effect of Time on Root Coverage Outcomes: A Network Meta-Analysis, Journal of Dental Research. (2019) 98, no. 11, 1195–1203, 10.1177/0022034519867071, 31381868. 31381868

[8] Barootchi S. , Tavelli L. , Di G. R. , Eber R. , Stefanini M. , Zucchelli G. , and Wang H. L. , Acellular Dermal Matrix for Root Coverage Procedures: 9-Year Assessment of Treated Isolated Gingival Recessions and Their Adjacent Untreated Sites, Journal of Periodontology. (2021) 92, no. 2, 254–262, 10.1002/JPER.20-0310, 32729954. 32729954

[9] Zabalegui I. , Sicilia A. , Cambra J. , Gil J. , and Sanz M. , Treatment of Multiple Adjacent Gingival Recessions With the Tunnel Subepithelial Connective Tissue Graft: A Clinical Report, International Journal of Periodontics & Restorative Dentistry. (1999) 19, no. 2, 199–206, 10635186. 10635186

[10] Allen A. L. , Use of the Supraperiosteal Envelope in Soft Tissue Grafting for Root Coverage. I. Rationale and Technique, International Journal of Periodontics & Restorative Dentistry. (1994) 14, no. 3, 216–227, 7995692. 7995692

[11] Tavelli L. , Barootchi S. , Nguyen T. V. N. , Tattan M. , Ravidà A. , and Wang H. L. , Efficacy of Tunnel Technique in the Treatment of Localized and Multiple Gingival Recessions: A Systematic Review and Meta-Analysis, Journal of Periodontology. (2018) 89, no. 9, 1075–1090, 10.1002/JPER.18-0066, 29761502. 29761502

[12] Chambrone L. and Tatakis D. N. , Periodontal Soft Tissue Root Coverage Procedures: A Systematic Review From the AAP Regeneration Workshop, Journal of Periodontology. (2015) 86, no. supplement 2, S8–S51, 10.1902/jop.2015.130674. 25644302

[13] Chambrone L. , Ortega M. A. S. , Sukekava F. , Rotundo R. , Kalemaj Z. , Buti J. , and Prato G. P. P. , Root Coverage Procedures for Treating Single and Multiple Recession-Type Defects: An Updated Cochrane Systematic Review, Journal of Periodontology. (2019) 90, no. 12, 1399–1422, 10.1002/JPER.19-0079, 31361330. 31361330

[14] Di Domenico G. , Guglielmi D. , Aroca S. , and de Sanctis M. , Treatment of Multiple Adjacent Gingival Recessions With a Coronally Advanced Flap vs a Modified Coronally Advanced Tunnel With a Volumetrically Stable Collagen Matrix: A 12-Month Randomized Controlled Clinical Trial, International Journal of Periodontics & Restorative Dentistry. (2024) 44, no. 5, 498–509, 10.11607/prd.6796, 37471160. 37471160

[15] Barootchi S. , Tavelli L. , Di Gianfilippo R. , Shedden K. , Oh T. J. , Rasperini G. , Neiva R. , Giannobile W. V. , and Wang H. L. , Soft Tissue Phenotype Modification Predicts Gingival Margin Long-Term (10-Year) Stability: Longitudinal Analysis of Six Randomized Clinical Trials, Journal of Clinical Periodontology. (2022) 49, no. 7, 672–683, 10.1111/jcpe.13641, 35561034. PMC9325391 35561034

[16] Hürzeler M. B. and Weng D. , A Single-Incision Technique to Harvest Subepithelial Connective Tissue Grafts From the Palate, International Journal of Periodontics & Restorative Dentistry. (1999) 19, no. 3, 279–287, 10635174. 10635174

[17] Tavelli L. , Barootchi S. , Di Gianfilippo R. , Modarressi M. , Cairo F. , Rasperini G. , and Wang H. L. , Acellular Dermal Matrix and Coronally Advanced Flap or Tunnel Technique in the Treatment of Multiple Adjacent Gingival Recessions. A 12-Year Follow-Up From a Randomized Clinical Trial, Journal of Clinical Periodontology. (2019) 46, no. 9, 937–948, 10.1111/jcpe.13163. 31242333

[18] Bertl K. , Melchard M. , Pandis N. , Müller-Kern M. , and Stavropoulos A. , Soft Tissue Substitutes in Non-Root Coverage Procedures: A Systematic Review and Meta-Analysis, Clinical Oral Investigations. (2017) 21, no. 2, 505–518, 10.1007/s00784-016-2044-4, 28108833. PMC5318480 28108833

[19] Tavelli L. , Barootchi S. , Stefanini M. , Zucchelli G. , Giannobile W. V. , and Wang H. L. , Wound Healing Dynamics, Morbidity, and Complications of Palatal Soft-Tissue Harvesting, Periodontology 2000. (2023) 92, no. 1, 90–119, 10.1111/prd.12466, 36583690. 36583690

[20] Stuhr S. , Nör F. , Gayar K. , Couso-Queiruga E. , Chambrone L. , Gamborena I. , Kumar P. , Avila-Ortiz G. , and Ganesan S. M. , Histological Assessment and Gene Expression Analysis of Intra-Oral Soft Tissue Graft Donor Sites, Journal of Clinical Periodontology. (2023) 50, no. 10, 1360–1370, 10.1111/jcpe.13843, 37424138. 37424138

[21] Mazzotti C. , Mounssif I. , Rendón A. , Mele M. , Sangiorgi M. , Stefanini M. , and Zucchelli G. , Complications and Treatment Errors in Root Coverage Procedures, Periodontology 2000. (2023) 92, no. 1, 62–89, 10.1111/prd.12468, 36594482. 36594482

[22] Chambrone L. , Pannuti C. M. , Tu Y. , and Chambrone L. A. , Evidence-Based Periodontal Plastic Surgery. II. An Individual Data Meta-Analysis for Evaluating Factors in Achieving Complete Root Coverage, Journal of Periodontology. (2012) 83, no. 4, 477–490, 10.1902/jop.2011.110382, 21859324. 21859324

[23] Gennai S. , Ben G. Z. , Orsolini C. , and Gosset M. , The Influence of Non-Carious Lesions in the Surgical Treatment of Gingival Recession: A Systematic Review & Meta-Analysis, Journal of Dentistry. (2022) 117, 103922, 10.1016/j.jdent.2021.103922, 34906618. 34906618

[24] Cairo F. , Barootchi S. , Tavelli L. , Barbato L. , Wang H. L. , Rasperini G. , Graziani F. , and Tonetti M. , Aesthetic-and Patient-Related Outcomes Following Root Coverage Procedures: A Systematic Review and Network Meta-Analysis, Journal of Clinical Periodontology. (2020) 47, no. 11, 1403–1415, 10.1111/jcpe.13346, 32654220. 32654220

