# Supplementary material for: Ten‐Year Split‐Mouth Follow‐Up of Tunnel Technique Using Connective Tissue Graft Versus Acellular Dermal Matrix for Multiple Gingival Recession Coverage: A Case Report
Source: Case Rep Dent. 2026 Oct 23;2026:1995303. doi: 10.1155/crid/1995303 (PMC13692707; doi:10.1155/crid/1995303)
Supplement: Supplementary file 1 [file CRID-2026-1995303-s001.pdf]

CARE case report guidelines

CARE Checklist of information to include when writing a case report

(cc) BY-NC-ND

Topic	Item	Checklist item description	Reported on Line
Title	1	The diagnosis or intervention of primary focus followed by the words "case report"	L1-3
Key Words	2	2 to 5 key words that identify diagnoses or interventions in this case report, including "case report"	L46-47
Abstract (no references)	3a	Introduction: What is unique about this case and what does it add to the scientific literature?	L24-29
	3b	Main symptoms and/or important clinical findings	L30-31; L35-40
	3c	The main diagnoses, therapeutic interventions, and outcomes	L30-40
	3d	Conclusion—What is the main "take-away" lesson(s) from this case?	L41-44
Introduction	4	One or two paragraphs summarizing why this case is unique (**may include references**)	L80-91
Patient Information	5a	De-identified patient specific information.	L95-L97
	5b	Primary concerns and symptoms of the patient.	L95-97
	5c	Medical, family, and psycho-social history including relevant genetic information	L95; L105-107
	5d	Relevant past interventions with outcomes	L105-107; L154-156
Clinical Findings	6	Describe significant physical examination (PE) and important clinical findings.	L94-105; L110-128
Timeline	7	Historical and current information from this episode of care organized as a timeline	L113-118; L150-187; L435-451
Diagnostic Assessment	8a	Diagnostic testing (such as PE, laboratory testing, imaging, surveys).	L103-105; L110-128
	8b	Diagnostic challenges (such as access to testing, financial, or cultural)	N/A
	8c	Diagnosis (including other diagnoses considered)	L94-98
	8d	Prognosis (such as staging in oncology) where applicable	N/A
Therapeutic Intervention	9a	Types of therapeutic intervention (such as pharmacologic, surgical, preventive, self-care)	L98-103; L130-170
	9b	Administration of therapeutic intervention (such as dosage, strength, duration)	L130-147;L150-170
	9c	Changes in therapeutic intervention (with rationale)	L150-156; L212-217
Follow-up and Outcomes	10a	Clinician and patient-assessed outcomes (if available)	L192-231
	10b	Important follow-up diagnostic and other test results	L192-217; L224-231; L435-451
	10c	Intervention adherence and tolerability (How was this assessed?)	L145-149; L169-177
	10d	Adverse and unanticipated events	L212-217
Discussion	11a	A scientific discussion of the strengths AND limitations associated with this case report	L228-248; L262-265; L281-295
	11b	Discussion of the relevant medical literature **with references**.	L238-254; L260-261;L271-280
	11c	The scientific rationale for any conclusions (including assessment of possible causes)	L244-265; L281-287
	11d	The primary "take-away" lessons of this case report (without references) in a one paragraph conclusion	L289-295
Patient Perspective	12	The patient should share their perspective in one to two paragraphs on the treatment(s) they received	L218-222
Informed Consent	13	Did the patient give informed consent? Please provide if requested	Yes ☒ No ☐ L301-305
